# Implantation of muscle satellite cells overexpressing myogenin improves denervated muscle atrophy in rats

**DOI:** 10.1590/1414-431X20155124

**Published:** 2016-02-05

**Authors:** H. Shen, Y. Lv, X.Q. Shen, J.H. Xu, H. Lu, L.C. Fu, T. Duan

**Affiliations:** 1 Department of Hand Surgery and Microsurgery Center, The First Affiliated Hospital, College of Medicine, Zhejiang University, Hangzhou, Zhejiang, China; 2 The Children's Hospital, Zhejiang University School of Medicine, Hangzhou, Zhejiang, China; 3 Department of Plastic Surgery, The First Affiliated Hospital, College of Medicine, Zhejiang University, Hangzhou, Zhejiang, China; 4 Toxicology Laboratory, College of Medicine, Zhejiang University, Hangzhou, Zhejiang, China

**Keywords:** Denervation, Muscle atrophy, Satellite cells, Myogenin, Transplantation, Transfection

## Abstract

This study evaluated the effect of muscle satellite cells (MSCs) overexpressing myogenin (MyoG) on denervated muscle atrophy. Rat MSCs were isolated and transfected with the MyoG-EGFP plasmid vector GV143. MyoG-transfected MSCs (MTMs) were transplanted into rat gastrocnemius muscles at 1 week after surgical denervation. Controls included injections of untransfected MSCs or the vehicle only. Muscles were harvested and analyzed at 2, 4, and 24 weeks post-transplantation. Immunofluorescence confirmed MyoG overexpression in MTMs. The muscle wet weight ratio was significantly reduced at 2 weeks after MTM injection (67.17±6.79) compared with muscles injected with MSCs (58.83±5.31) or the vehicle (53.00±7.67; t=2.37, P=0.04 and t=3.39, P=0.007, respectively). The muscle fiber cross-sectional area was also larger at 2 weeks after MTM injection (2.63×10^3^±0.39×10^3^) compared with MSC injection (1.99×10^3^±0.58×10^3^) or the vehicle only (1.57×10^3^±0.47×10^3^; t=2.24, P=0.049 and t=4.22, P=0.002, respectively). At 4 and 24 weeks post-injection, the muscle mass and fiber cross-sectional area were similar across all three experimental groups. Immunohistochemistry showed that the MTM group had larger MyoG-positive fibers. The MTM group (3.18±1.13) also had higher expression of MyoG mRNA than other groups (1.41±0.65 and 1.03±0.19) at 2 weeks after injection (t=2.72, P=0.04). Transplanted MTMs delayed short-term atrophy of denervated muscles. This approach can be optimized as a novel stand-alone therapy or as a bridge to surgical re-innervation of damaged muscles.

## Introduction

Peripheral nerve injury-induced muscle atrophy has a complex and multifactorial pathophysiology. Muscle re-innervation is frequently insufficient to regain lost muscle strength, and functional benefits remain limited (1). Strategies to decrease detrimental muscle changes after denervation include cell-based therapies (2 3 4). Transplanted Schwann cells and adipose-derived stem cells might reduce muscle atrophy and benefit patients with nerve injury (3). Transplanted muscle satellite cells (MSCs) self-renew vigorously and repopulate host muscles (5). In this study, we evaluated the effect of transplanting MSCs that overexpress the myogenic protein myogenin (MyoG) on muscle atrophy development in a rodent muscle denervation model.

MyoG is essential for the development of functional skeletal muscle (6). Following muscle denervation, MyoG expression is upregulated in affected muscles within 7 days, but high expression levels are not maintained beyond 1 month (7). Atrophy of the denervated muscle is thought to be prevented by MyoG expression (8). Denervation not only induces muscle myogenin expression but also enhances MSC activity in muscle tissues (9). There is a myogenic response by MSCs during the first 3-4 weeks after acute denervation to replace lost muscle fibers (10). MSCs stimulate myogenic cell activity, and these cell types work together to regenerate damaged muscles (11). Transfection of the MyoG gene might enhance regeneration of denervated muscles. Although direct transfection of MyoG into the gastrocnemius muscle has been reported, its effects on muscle physiology are inconsistent (12). Implantation of cultured MSCs has proved to be efficient to inhibit denervation muscle atrophy (13). We hypothesized that transplanting MSCs that overexpress MyoG would additionally reduce post-denervation muscle atrophy.

## Material and Methods

### Isolation and culture of skeletal MSCs

The experimental protocol was approved by the Institutional Animal Care and Use Committee. Specific pathogen-free Sprague Dawley rats (male, 8 weeks old, 160-180 g) were euthanized by pentobarbital injection. Hind limb muscles were blunt dissected and aseptically cut into <1 mm^3^ pieces. Muscle fragments were cultured in six-well plates in high-glucose Dulbecco's modified Eagle's medium (Invitrogen, USA) containing 15% fetal bovine serum (Invitrogen), 100 IU/mL penicillin, and 100 IU/mL streptomycin at 37°C in a 5% CO_2_ and 95% humidity incubator. Outgrowing fibroblasts were segregated by differential cell adhesion after 2 h of explant culture (14). The collected cell population, which primarily consisted of MSCs, was transferred to a new 5-cm culture dish for *in vitro* expansion.

### Overexpression of MyoG in MSCs

The MyoG-EGFP construct encoded both MyoG and enhanced green fluorescent protein. The coding sequence of the rat MyoG gene (GenBank Accession No. NM_017115) was subcloned into the *Xho* I and *Kpn* I sites of the plasmid vector GV143 (Shanghai Genechem). At 80% confluence, cells were transfected using X-tremeGENE HP DNA transfection reagent (Roche, Switzerland). Cells transfected with the empty vector were used as negative controls to determine the MyoG-specific transfection efficacy. Laser scanning confocal microscopy (BX61W1-FV1000; Olympus, Japan) was used to detect immunofluorescence in MyoG-transfected MSCs (MTMs). A mouse anti-MyoG monoclonal antibody (M5815; Sigma-Aldrich, USA) was used at a 1:100 dilution for immunostaining. Nuclear staining was performed using 4',6'-diamidino-2-phenylindole dihydrochloride (100 nM; Sigma).

### Denervation and MSC injection

To establish the sciatic denervation model, the sciatic nerve was cut in pentobarbital-anaesthetized rats and a 1 cm segment was excised. At 1 week after denervation, 200 µL phosphate-buffered saline (PBS) containing 1×10^6^ suspended MTMs was injected into the medial gastrocnemius muscle through a 0.5-cm incision. Two control groups were injected with either 1×10^6^ untransfected MSCs in suspension or the vehicle only, in 200-µL volumes. Each group included six rats. At 2, 4, and 24 weeks after injection, the medial gastrocnemius muscle was excised from the animals and processed for immunostaining and PCR analyses.

### Immunohistochemical analyses

Immunohistochemical staining of formalin-fixed, paraffin-embedded muscle sections for MyoG was performed using a mouse monoclonal anti-MyoG antibody (1:100, M5815; Sigma). The cross-sectional muscle fiber area, an index of muscle atrophy, was analyzed in hematoxylin and eosin (H&E)-stained tissue sections using Image J 2.1 software.

### RNA extraction and real-time PCR

To measure MyoG gene expression, the GenBank-retrieved sequence was used to design the following MyoG primers for PCR: forward, 5'-GGCAATGCACTGGAGTTTGG-3'; reverse, 5'-CAATCTCAGTTGGGCATGGTTTC-3'. Glyceraldehyde-3-phosphate dehydrogenase (GAPDH) was used as the internal reference with the following primers: forward, 5'-CAAGCATCCATGCGGGAAC-3'; reverse, 5'-ATGGTGGTGAAGACGCCAGTA-3'. Primers for real-time PCR were obtained from Takara Bio (Japan). First-strand cDNA synthesis was performed using 500 ng total RNA and PrimeScript™ RT Master Mix (Japan). Real-time PCR was performed on a LightCycler 480 system using SYBR Premix Ex Taq II (Japan) and 200 nM of each primer in a 10 µL reaction volume. Results are reported as fold changes in MyoG gene expression calculated by the 2^-ΔΔCT^ method.

### Statistical analysis

Statistical analyses were performed using SPSS 16.0 (SPSS Inc., USA). Data are reported as means±SD. Means were compared between groups by the two-tailed *t*-test. P<0.05 was considered to be statistically significant.

## Results

### Expression of MyoG in MTMs

**Figure 1:**
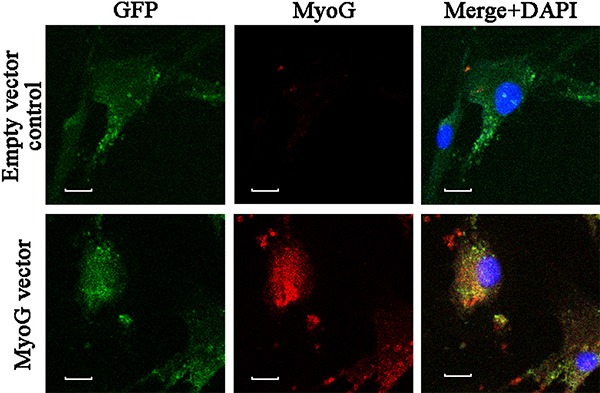
Expression of MyoG in transfected MSCs. Representative images of immunocytochemistry showing green fluorescent protein (green) in empty vector-transfected MSCs, whereas MyoG was clearly expressed (red) in MTMs. Nuclei were stained with 4',6'-diamidino-2-phenylindole dihydrochloride (DAPI; blue). Scale bars=10 µm. MyoG: myogenin; MSCs: muscle satellite cells; GFP: green fluorescent protein.

Primary MSC cultures were used at passages 2-4. Fluorescence detection of green fluorescent protein indicated that both transfectants (vector only and the vector containing the MyoG sequence) had incorporated the plasmid. Cells transfected with the MyoG sequence displayed a strong MyoG-specific immunofluorescent signal, whereas cells transfected with the empty vector showed a weak background signal (Figure 1). Incubation with primary or secondary antibodies alone resulted in no signal, supporting the specificity of the antibodies.

### MTM implantation maintained the muscle mass and fiber size in denervated muscles

**Figure 2:**
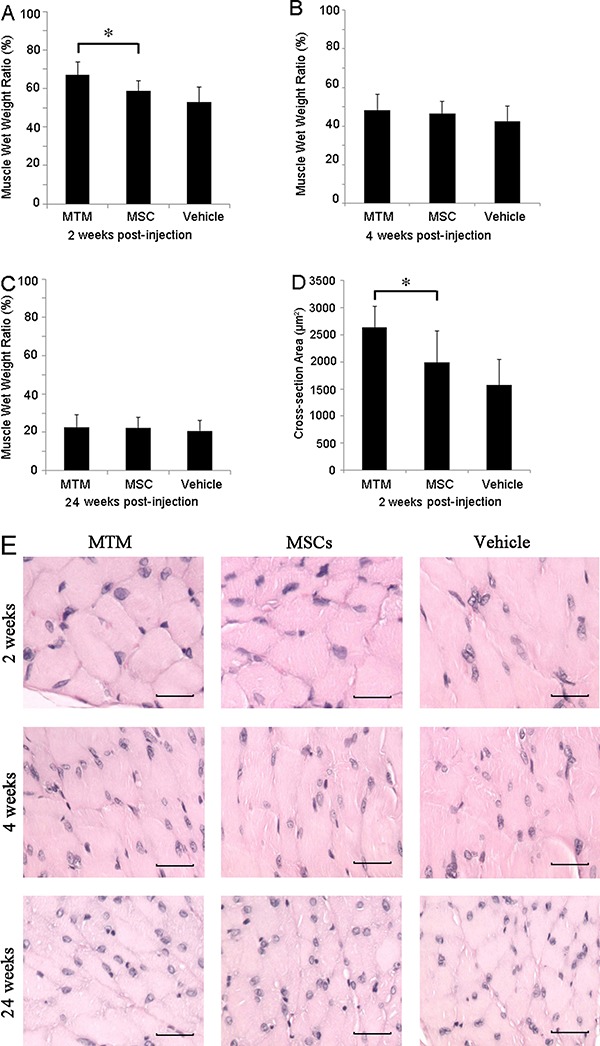
Changes in the denervated muscle mass and fiber size after MTM transplantation. *A*, At 2 weeks after MTM injection into denervated rat gastrocnemius muscles, animals displayed less muscle mass loss than animals injected with empty vector-transfected cells or the vehicle only (*t=0.39, P=0.007 and t=2.37, P=0.04, respectively). *B* and *C,* no significant differences were found in muscle masses across all three experimental groups at 4 and 24 weeks after cell transplantation. *D* and *E*, The average cross-sectional muscle fiber area at 2 weeks post-injection was significantly larger in MTM-injected animals than in animals injected with empty vector-transfected MSCs or the vehicle only (*t=4.22, P=0.002 and t=2.24, P=0.049, respectively). *E,* There was no difference in the muscle fiber cross-sectional area at 4 and 24 weeks post-injection. Data are reported as means±SD of the ratios of measurements in denervated *vs* contralateral non-operated muscles of each animal. More comprehensive data analyses of the muscle mass and fiber cross-sectional area are provided in Supplementary Tables S1 and S2. MTMs, MyoG-transfected muscle satellite cells; MSCs, muscle satellite cells; MyoG, myogenin.

All denervated gastrocnemius samples lost wet muscle mass over time compared with the unoperated contralateral muscle. The ratio of operated *vs* unoperated contralateral muscle wet weights was significantly higher in MTM-transplanted animals than in MSC and vehicle control groups at 2 weeks post-injection (Figure 2A-C, Supplementary Table S1). There was no significant difference in muscle mass loss in the operated leg between MTM-transplanted animals and untransfected MSC or vehicle control-injected animals at 4 and 24 weeks post-injection.

H&E staining showed that the gastrocnemius muscle fibers in the MTM group had significantly larger cross-sectional areas than the other groups at 2 weeks post-injection (Figure 2D, Supplementary Table S2). This difference was not detectable in all three groups at 4 and 24 weeks (Figure 2E), because cytolysis and atrophy of the muscle fibers at these time points eliminated distinctive inter-fiber anatomical boundaries, making fiber size analysis impossible.

### MTM implantation temporarily increased the number of MyoG+ fibers in denervated gastrocnemius muscles

**Figure 3:**
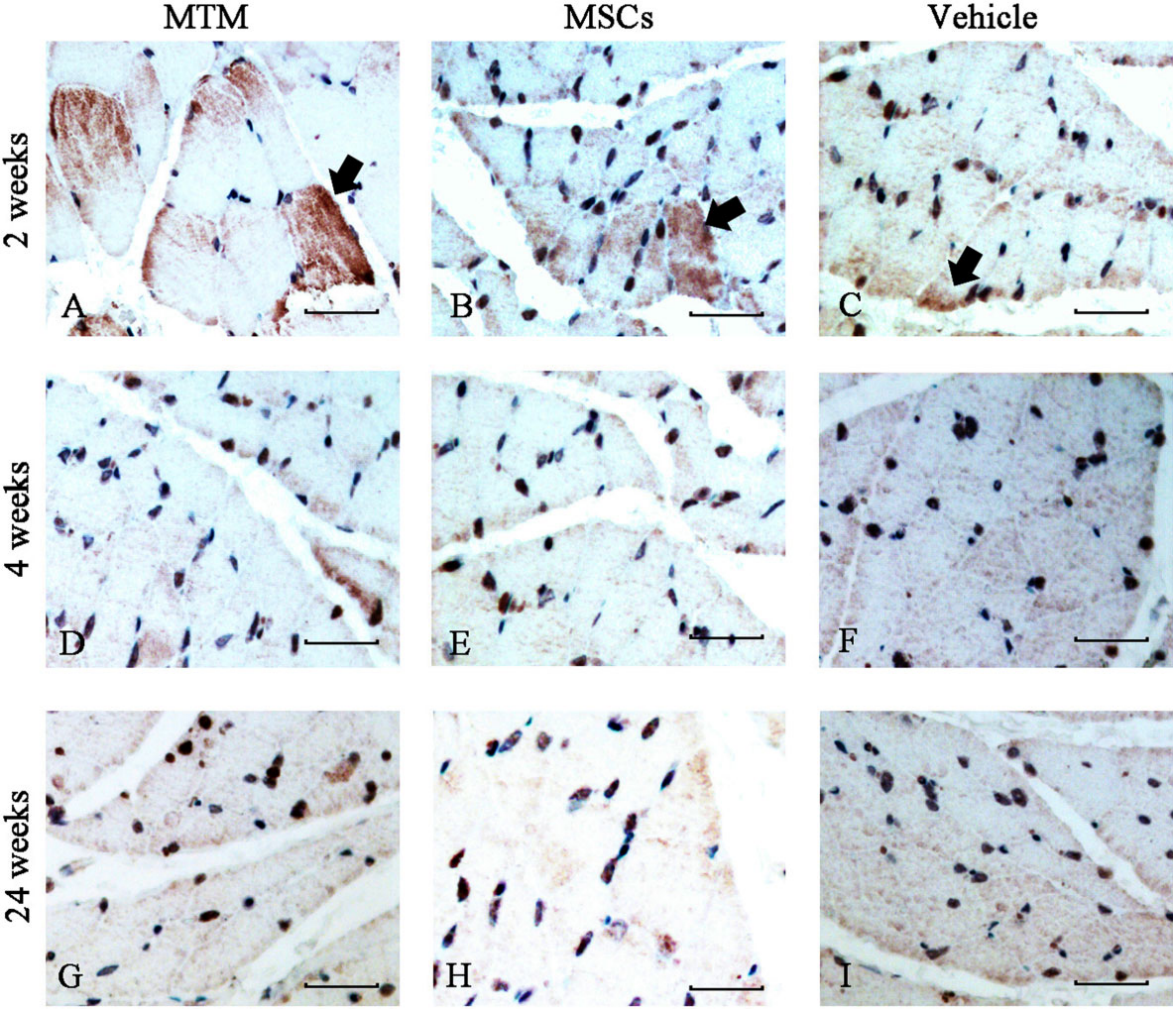
Muscle fiber anatomy and MyoG protein expression in denervated rat muscles after MTM transplantation. MyoG-positive muscle fibers were stained with antibodies against ciliary neurotrophic factor (CNTF) and MyoG (brown). *A-C*, At 2 weeks after cell injection, MyoG-positive muscle fibers were more prominent in denervated gastrocnemius muscles of the MyoG overexpression group (arrows). *D-I*, At 4 and 24 weeks after treatment, MyoG-positive muscle fibers displayed less clear boundaries because of extensive cytolysis. Scale bars=50 µm. MTMs: MyoG-transfected muscle satellite cells; MyoG: myogenin.

Immunohistochemical analysis revealed that muscle tissue sections from the MTM group contained more MyoG **^+^** fibers than the other groups at 2 weeks post-injection. There was no significant difference in the fiber number of the MSC group compared with the vehicle control group at 2 weeks post-injection. At 4 and 24 weeks post-injection, all three groups showed similar decreases in the numbers of MyoG **^+^** fibers as well as extensive cytolysis and collagen hyperplasia in the denervated gastrocnemius (Figure 3).

### MyoG mRNA expression was temporarily increased in denervated gastrocnemius muscles injected with MTMs

**Figure 4:**
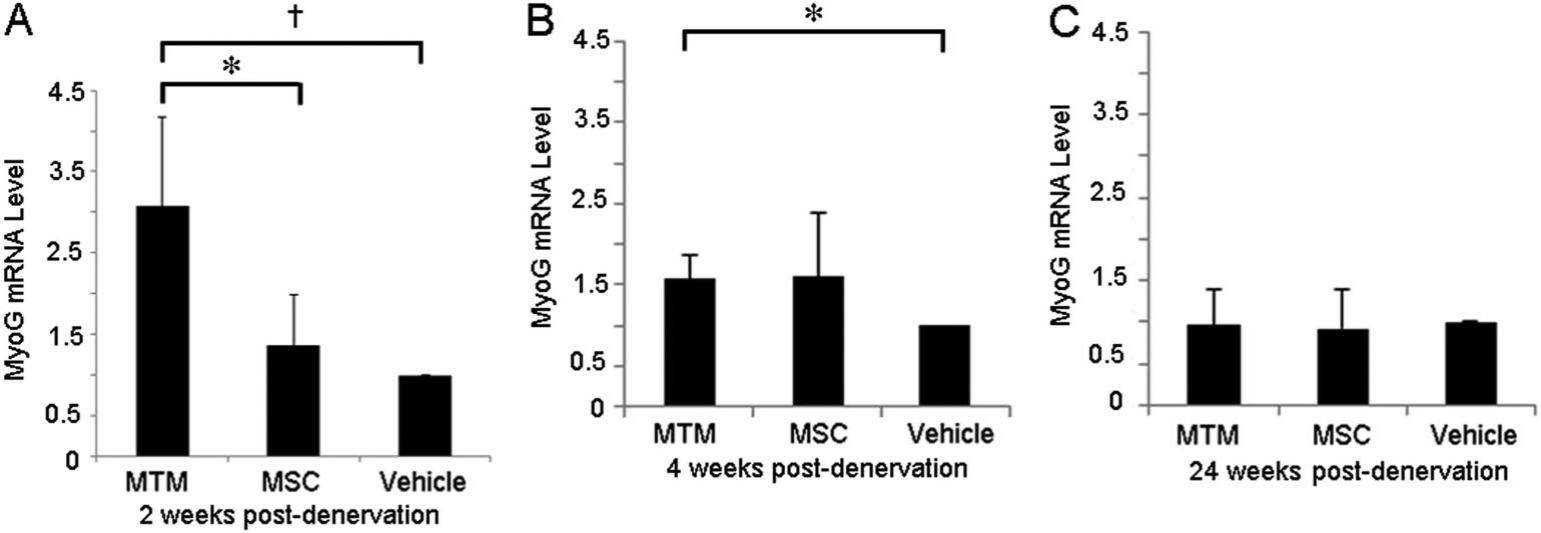
MyoG mRNA expression in denervated rat gastrocnemius muscles. *A*, Implantation of MTMs overexpressing MyoG increased MyoG mRNA levels compared with untransfected MSCs and the vehicle control at 2 weeks post-injection. (*t=2.72, P=0.04 and ^†^t=3.75, P=0.01, respectively). *B* and *C*, MyoG mRNA levels were decreased at 4 weeks in all groups, although the MyoG level in the MTM group remained significantly higher than that in the vehicle control group (*t=2.82, P=0.03). There were no significant differences in MyoG expression across all three experimental groups at 24 weeks after injection. Data are reported as means±SD. Detailed quantitative values are provided in Supplementary Table S3. MyoG: myogenin; MTMs: MyoG-transfected muscle satellite cells; MSCs: muscle satellite cells.

Implantation of MSCs overexpressing MyoG significantly increased gastrocnemius expression of MyoG mRNA (3.18±1.13 ratio to GAPDH expression) compared with gastrocnemius muscles injected with untransfected MSCs or the vehicle only (1.41±0.65 and 1.03±0.19, respectively) at 2 weeks post-injection. At 4 weeks post-transplantation, MyoG expression was significantly reduced in all groups, but remained comparatively elevated in the MTM group (0.44±0.08 ratio) compared with the vehicle control group (0.27±0.09) (Figure 4, Supplementary Table S3).

## Discussion

Transplantation of MyoG-overexpressing MSCs delayed muscle atrophy in a rat model of skeletal muscle denervation. MTM-transplanted muscles showed significant increases in MyoG expression with a concurrent decreased rate of muscle mass loss and a better-maintained muscle fiber cross-sectional area for at least 2 weeks after MTM injection. These effects of MTMs on muscle degeneration were transient, and no differences in the outcome parameters were detected at 4 and 24 weeks post-injection. Injection of untransfected MSCs also temporarily improved denervated muscle injury parameters. However, simultaneously induced MyoG overexpression provided significant additional benefits. Thus, MSCs and elevated MyoG expression function in an additive fashion to alleviate atrophy of denervated muscles.

MSCs have a clear ability to generate differentiated, multi-nucleated muscle fibers *in vitro* and *in vivo* (15,16). In addition, MSCs maintain their own functional population by self-renewal. Implantation of a single myofiber with MSCs has indicated that MSCs are capable of expanding their own population to repopulate irradiated MSC-depleted muscles (5). However, implantation of freshly-isolated, enzymatically-disaggregated MSCs alone results in poor muscle regeneration (17,18). In contrast, implantation of cultured MSCs *in vitro* has proved to be efficient (13,19). In the current study, we obtained similar results.

MyoG has skeletal muscle differentiation-inducing effects that cannot be replaced by other myogenic regulatory factors (20). MyoG participates in the formation of new myotubes. Although muscle atrophy progression is most obvious at 2-4 weeks following muscle denervation, MyoG expression is upregulated in skeletal muscles within 12 h of denervation injury (21) with expression levels peaking by 7 days but not maintained beyond 1 month (7). This time course of injury-induced MyoG expression parallels the temporal MyoG expression profile in the present study. However, a single injection of MyoG-overexpressing MSCs was not sufficient to maintain elevated MyoG levels beyond 4 weeks. These observations support the consensus that therapeutic re-innervation should be performed as soon as possible after injury.

To avoid the acute phase of inflammation, a recent study on neural stem cell implantation has indicated that the optimal time point for implantation is 1 week after denervation injury (22). Thus, we designed our time course experiments accordingly. Conversely, maximal MSC responses to muscle denervation occur within the first 3-4 weeks after acute injury, resulting in replacement of lost fibers. From 7 to 20 weeks post-denervation, MSC numbers and functions decline with concurrent increases in fibrosis and adipose cell expansion (3,10). In histological and gene expression analyses, the additional early regenerative activity of injected MSCs and elevated MyoG expression had decreased by 4 weeks after implantation. Therefore, surgical re-innervation should occur within that time frame to improve the outcome. Furthermore, it may be beneficial to administer a second stem cell injection during the early post-injury period. In a rabbit denervation model, cell therapy improved the properties of denervated muscles only when they were allowed to re-innervate, which is in contrast to our results (23). However, positive findings have been observed after rat adipose-derived stem cell implantation without re-innervation (3).

The mechanisms underlying MSC-improved denervation injury remain to be clarified in further research. In this study, we transplanted cultured MSCs, but the influence of *in vitro* culture and transfection on cellular activity is unclear. Transplanted MSCs may be able to self-renew and then return to a quiescent state (5). It is also important to determine whether the transfected MSCs maintain their overexpression of MyoG.

Implantation of MTMs delays denervated muscle atrophy, at least in the short term. MSC implantation combined with gene therapy may have a therapeutic utility in prevention of denervated muscle atrophy, either as a stand-alone treatment or as a bridge to surgical re-innervation.

## Supplementary Material


