# Supplementary material for: Implantation of muscle satellite cells overexpressing myogenin improves denervated muscle atrophy in rats
Source: Braz J Med Biol Res. 2016 Feb 5;49(2):e5124. doi: 10.1590/1414-431X20155124 (PMC4742975; doi:10.1590/1414-431X20155124)
Supplement: Supplementary file 1 [file 1414-431X-bjmbr-1414-431X20155124-S1.pdf]

Table S1. Wet muscle weight ratio (%).

	MTMs	MSCs	Control	P
2 weeks	67.17±6.79	58.83±5.31	53.00±7.67	0.04^{+}<br>0.007$^{\#}$<br>0.16$^{\&}$
4 weeks	48.17±8.21	46.50±6.41	42.50±7.92	0.70^{+}<br>0.25$^{\#}$<br>0.36$^{\&}$
24 weeks	22.83±6.52	22.17±5.74	20.67±5.50	0.86^{+}<br>0.55$^{\#}$<br>0.65$^{\&}$

Data are reported as means±SD. MTMs: MyoG-transfected MSCs; MSCs: muscle satellite cells. $^{+}$MTMs vs MSCs; $^{\#}$MTMs vs vehicle control; $^{\&}$MSCs *vs* vehicle control (Student's t-test).

Table S2. Cross-sectional area of muscle fibers (μm^2).

	MTMs	MSCs	Control	P
2 weeks	2.63×103±0.39×103	1.99×103±0.58×103	1.57×103±0.47×103	0.049+<br>0.002$^{\#}$<br>0.21$^{\&}$

MTMs: MyoG-transfected MSCs; MSCs: muscle satellite cells. $^{+}$MTMs *vs* MSCs; $^{\#}$MTMs *vs* vehicle control; $^{\&}$MSCs *vs* vehicle control (Student's *t*-test).

Table S3. Expression levels of MyoG normalized to GADPH levels ($2^{-\Delta\Delta CT}$).

	MTMs	MSCs	Control	P
2 weeks	3.18±1.13	1.41±0.65	1.03±0.19	0.04^{+}<br>0.01$^{\#}$<br>0.31$^{\&}$
4 weeks	0.44±0.08	0.44±0.22	0.27±0.09	0.98^{+}<br>0.03$^{\#}$<br>0.21$^{\&}$
24 weeks	0.23±0.10	0.22±0.11	0.24±0.08	0.90^{+}<br>0.89$^{\#}$<br>0.79$^{\&}$

MTMs: MyoG-transfected MSCs; MSCs: muscle satellite cells. $^{+}$MTMs *vs* MSCs; $^{\#}$MTMs *vs* vehicle control; $^{\&}$MSCs *vs* vehicle control (Student's *t*-test).
